# Assessment of Pulpal Oxygen Saturation in Healthy and Diseased Primary Teeth Using a Customized Pulse Oximeter Holder in Children Aged Four to Nine Years: A Cross-Sectional Study

**DOI:** 10.7759/cureus.114464

**Published:** 2026-08-13

**Authors:** Snehal Jagtap, Nupur Ninawe, Ritesh Kalaskar, Rakesh Bahadure, Naveen Reddy Banda, Utkarsha Kadam

**Affiliations:** 1 Department of Paediatric and Preventive Dentistry, Government Dental College and Hospital, Nagpur, IND; 2 Department of Paediatric Dentistry, Ibn Sina National College, Jeddah, SAU

**Keywords:** customized pulse oximeter holder, dental pulp diseases, diagnostic techniques, pediatric dentistry, primary teeth, pulp vitality, pulse oximetry

## Abstract

Introduction

Accurate assessment of pulp vitality is essential for diagnosis and treatment planning in pediatric dentistry. Conventional pulp sensibility tests, including thermal and electric pulp testing, assess neural response rather than pulpal vascularity and often demonstrate limited reliability in primary teeth. Pulse oximetry provides a non-invasive, objective assessment of pulpal oxygen saturation; however, evidence regarding its use in primary dentition remains limited. This study evaluated pulpal oxygen saturation in healthy and diseased primary teeth using a customized pulse oximeter holder and established reference values for different tooth types and pulpal conditions.

Methods

This descriptive cross-sectional study included 610 primary teeth from 271 children aged four to nine years, with multiple teeth included from some participants. Phase I comprised 530 healthy primary teeth, including maxillary and mandibular incisors, canines, first molars, and second molars. Phase II included 80 mandibular primary molars categorized as healthy pulp, reversible pulpitis, irreversible pulpitis, pulpal necrosis, and endodontically treated teeth (n = 16/group). A customized three-dimensional printed pulse oximeter holder was fabricated to ensure optimal adaptation and parallel alignment of the light-emitting diode (LED) and photodetector. Pulpal diagnosis was established using clinical history, clinical and radiographic examination, cold testing, electric pulp testing, and bleeding on access cavity preparation when indicated. Pulpal oxygen saturation was recorded three times for each tooth, and the mean value was analyzed. Statistical significance was set at p < 0.05.

Results

Pulpal oxygen saturation differed significantly among primary tooth types in both arches (p < 0.001). Maxillary central incisors exhibited the highest mean oxygen saturation (89.29 ± 2.20%), whereas mandibular second molars demonstrated the lowest values among healthy teeth (84.18 ± 2.81%). Anterior teeth showed significantly higher oxygen saturation than posterior teeth in both arches (p < 0.001). In Phase II, mean pulpal oxygen saturation progressively declined from healthy pulp (83.44 ± 2.56%) to reversible pulpitis (80.25 ± 2.70%), irreversible pulpitis (70.50 ± 3.98%), pulpal necrosis (11.13 ± 15.34%), and endodontically treated teeth (0.00%) (p < 0.001). Healthy pulp and reversible pulpitis demonstrated overlapping oxygen saturation values, whereas advanced pulpal disease showed significantly lower values. Finger oxygen saturation remained stable across all groups.

Conclusions

Pulpal oxygen saturation in primary teeth varied according to tooth type, dental arch, anatomical region, and pulpal status, demonstrating a progressive decline with advancing pulpal pathology. The customized pulse oximeter holder enabled consistent and reproducible measurements across different primary teeth. Pulse oximetry appears to be a promising objective and non-invasive adjunct to conventional pulp sensibility tests for assessing pulp vitality in children. However, further longitudinal studies incorporating appropriate statistical methods to account for clustered observations and larger study populations are needed to validate tooth-specific reference values and establish its routine clinical applicability.

## Introduction

Accurate endodontic diagnosis depends on a thorough understanding of pain physiology, detailed history taking, and systematic clinical examination. The nature, duration, and precipitating factors of pain, combined with extraoral and intraoral findings, form the foundation of a reliable diagnosis. Central to this process is the assessment of pulp status, which directly governs treatment planning decisions in pediatric dentistry [1].

Since the dental pulp is enclosed within hard tissues, direct evaluation is not feasible, and clinicians rely on indirect pulp vitality tests. Conventional methods, including thermal tests, electric pulp testing, and test cavity preparation, assess the sensory response of pulpal nerve fibers rather than true vascular perfusion and are therefore more accurately termed "pulp sensibility tests" [2]. These tests are inherently limited by their dependence on the patient's subjective perception of stimuli, making them prone to false-positive and false-negative responses. Furthermore, pulpal nerve fibers may remain responsive even after significant vascular compromise or, conversely, fail to respond despite an intact blood supply, thereby reducing diagnostic reliability [3].

Pulse oximetry offers a non-invasive, objective alternative by directly measuring arterial oxygen saturation based on differential light absorption by oxygenated and deoxygenated hemoglobin, entirely independent of neural activity [4]. Widely used in medical practice since its development by Takuo Aoyagi in the early 1970s, its dental application has been explored using customized probe holders, predominantly in permanent teeth [5, 6]. Janani et al. demonstrated the feasibility of a three-dimensionally printed pulse oximeter probe holder for pulpal oxygen saturation assessment in primary teeth, highlighting its potential as a clinically viable vitality assessment tool in pediatric patients [2].

Despite these promising findings, important gaps remain in the literature. Most published studies have evaluated only healthy primary teeth or a limited number of tooth types, with few investigating pulpal oxygen saturation across different pulpal conditions. Furthermore, reference oxygen saturation values for the full spectrum of primary tooth types and pulpal diagnoses have not been comprehensively established. This limits the clinical interpretation and wider application of pulse oximetry in primary dentition.

Reliable pulse oximetry also depends on accurate sensor positioning. The probe holder must maintain parallel alignment between the light-emitting diode and photodetector while adapting closely to tooth morphology to ensure consistent signal transmission and reproducible measurements. Therefore, a customized dental pulse oximeter holder was used in the present study to improve probe adaptation and measurement consistency across different primary teeth.

Accordingly, this cross-sectional study aimed to evaluate pulpal oxygen saturation in healthy and diseased primary teeth using a customized pulse oximeter holder, compare oxygen saturation values across different primary tooth types and pulpal conditions, and establish reference values that may support the objective assessment of pulp vitality in primary dentition.

## Materials and methods

The present descriptive cross-sectional study was conducted in the Department of Paediatric and Preventive Dentistry of Government Dental College and Hospital, Nagpur, India, from January 2025 to March 2026. Ethical approval was obtained from the Institutional Ethics Committee (Ref. No. IEC/GDCHN/79/2024), and the study was conducted in accordance with the principles of the Declaration of Helsinki, 1975.

Study population and sampling

Children aged four to nine years reporting to the department were screened for eligibility using a convenience sampling method. Parents or guardians of eligible participants were informed about the study purpose and procedures in their vernacular language, and written informed consent was obtained prior to enrollment. A total of 610 primary teeth from 271 children were included in the study. Depending on the eligibility criteria, some participants contributed more than one primary tooth. Therefore, the unit of analysis was the individual tooth. The inclusion and exclusion criteria are summarized in Table 1.

**Table 1:** Inclusion and exclusion criteria

Serial no	Inclusion Criteria	Exclusion Criteria
1	Children aged four to nine years with Frankl Behavioral Rating Scale scores of 3 and 4 [7]	Children with Frankl Behavioral Rating Scale scores of 1 and 2 [7]
2	Primary teeth with no clinical signs or symptoms of pulpal disease and no radiographic evidence of root resorption	Children with radiographic evidence of root resorption
3	Primary teeth with signs or symptoms suggestive of pulpal disease (dull aching, spontaneous, or nocturnal pain; dento-alveolar abscess; sinus or fistula) without radiographic evidence of root resorption	Children exhibiting tooth mobility
4	Children who had undergone endodontic treatment of primary molars	Children with systemic illness or special healthcare needs
5	Parents or guardians willing to provide written informed consent	Grossly decayed teeth with significant loss of crown structure
6	—	Parents or guardians unwilling to provide informed consent

Sample size

Sample size was estimated using two statistical approaches. For Phase I (healthy primary teeth), sample size was estimated using a pairwise comparison based on one-way ANOVA with mean oxygen saturation values reported by Grabliauskienė et al. [8]. The calculation indicated that 105 teeth were required for each pairwise comparison, corresponding to approximately 53 teeth per comparison group. Accordingly, 53 teeth were included in each of the 10 healthy primary tooth subgroups, resulting in a total sample size of 530 teeth.

For Phase II (molars across pulpal conditions), sample size was estimated using G*Power software (v3.1.9.7, Heinrich-Heine-Universität Düsseldorf, Düsseldorf, Germany) with a large effect size (f = 0.4) as suggested by Cohen, α = 0.05, power = 80%, and five groups, yielding a total of 80 teeth with 16 teeth per group.

Study groups

The study sample comprised 610 primary teeth and was divided into two phases based on the study objectives. Phase I included 530 healthy primary teeth distributed according to tooth type in the maxillary and mandibular arches to establish baseline pulpal oxygen saturation values. Phase II included 80 primary molars categorized into five groups based on pulpal status (healthy, reversible pulpitis, irreversible pulpitis, pulpal necrosis, and endodontically treated teeth) to evaluate pulpal oxygen saturation across different pulpal conditions. The distribution of the study groups is presented in Table 2.

**Table 2:** Distribution of the study sample according to study phases and groups

Study Phase	Category	Group	Description	Sample Size (n)
Phase I: Healthy primary teeth (n = 530)	Maxillary teeth	Group 1a	Central incisor	53
		Group 1b	Lateral incisor	53
		Group 1c	Canine	53
		Group 1d	First molar	53
		Group 1e	Second molar	53
	Mandibular teeth	Group 2a	Central incisor	53
		Group 2b	Lateral incisor	53
		Group 2c	Canine	53
		Group 2d	First molar	53
		Group 2e	Second molar	53
Phase II: Primary molars across pulpal conditions (n = 80)	Pulpal status	Group A	Healthy molars	16
		Group B	Reversible pulpitis	16
		Group C	Irreversible pulpitis	16
		Group D	Pulpal necrosis	16
		Group E	Endodontically Treated Molars	16
Overall	—	—	Total Study Sample	610

Development of the customized dental holder

**Figure 1:**
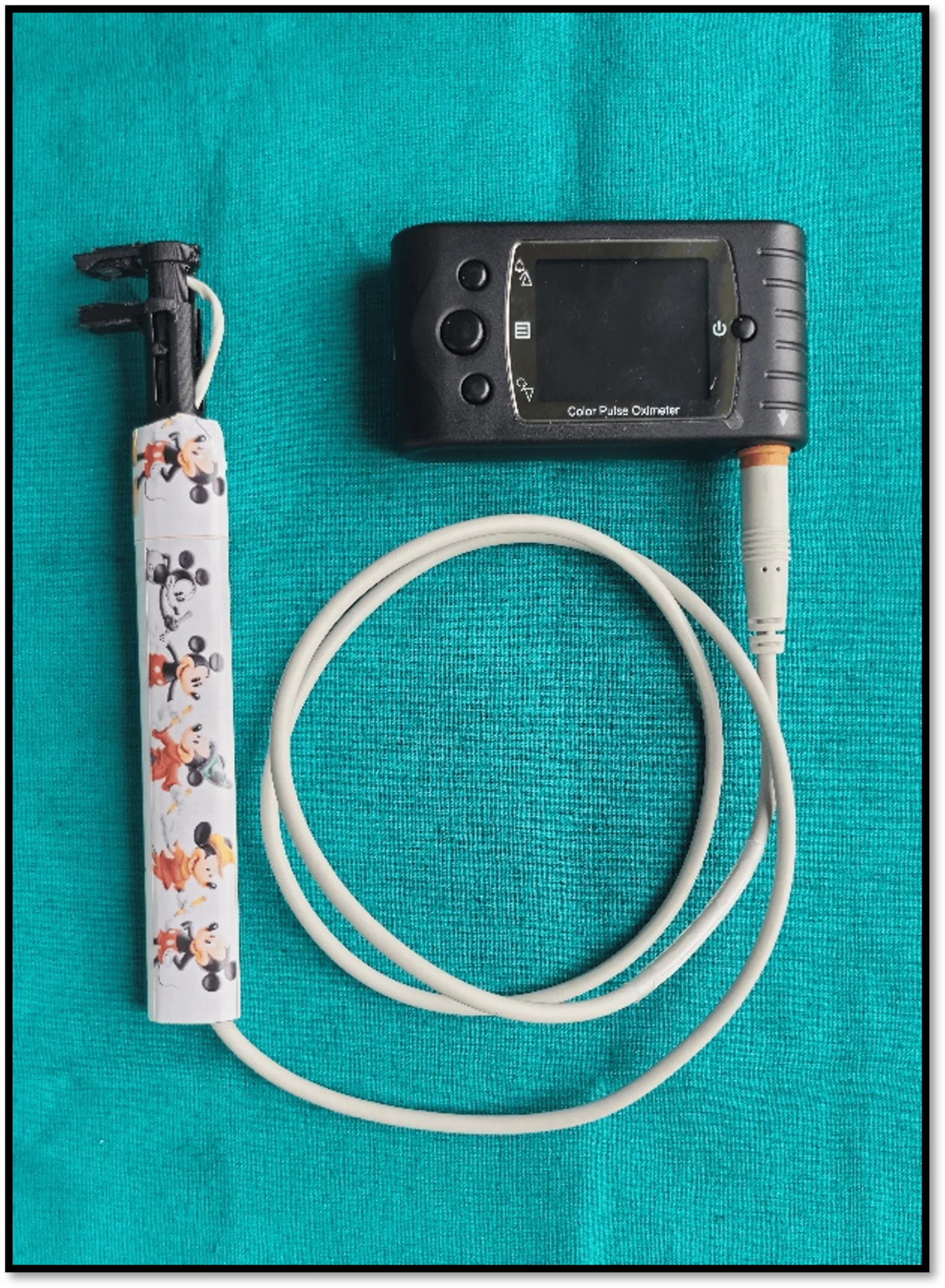
Device for measuring pulp oxygen saturation

A customized pulse oximeter holder was designed and fabricated to ensure reliable oxygen saturation measurements in primary teeth. Digital impressions of the primary dentition were obtained using an intraoral scanner (TRIOS, 3Shape, Copenhagen, Denmark) and exported in STL format. The holder was designed using computer-aided design (CAD) software (SolidWorks 2024, Dassault Systèmes SolidWorks Corp., Waltham, MA, USA), incorporating a sliding caliper mechanism to accommodate variations in the labiolingual dimensions of primary teeth while maintaining strict parallel alignment between the light-emitting diode (LED) and photodetector, a prerequisite for accurate and reproducible signal transmission. The customized holder was fabricated using a three-dimensional printer (Creality Ender-3 V3 SE, Shenzhen Creality 3D Technology Co., Ltd., Shenzhen, China) with polylactic acid (PLA) filament, selected for its biocompatibility and dimensional stability. The design and three-dimensional printing of the customized holder were carried out by MRO Engineering and Supplies Pvt. Ltd., Nagpur, Maharashtra, India. The LED and photodetector components were retrieved from a Contec™ CMS60C pulse oximeter (Contec Medical Systems Co., Ltd., Qinhuangdao, China) and integrated into the customized holder. The connecting wire was routed through a designated internal channel, maintaining connection to the main display unit (Figure 1).

Clinical procedure and pulpal diagnosis

Prior to assessment, oral prophylaxis was performed to minimize interference with oxygen saturation readings. Pulpal status was determined by integrating clinical history, intraoral examination, radiographic evaluation, cold testing using ethyl chloride refrigerant spray (Endo-Frost, Coltene, Switzerland), and electric pulp testing (Waldent Innovations Pvt. Ltd., New Delhi, India). Teeth were classified into four diagnostic categories: healthy pulp, reversible pulpitis, irreversible pulpitis, and pulpal necrosis using standardized predefined criteria. A healthy pulp demonstrated no symptoms, normal radiographic appearance, and a brief non-lingering response to sensibility tests. Reversible pulpitis presented with short, sharp pain with no radiographic pathology. Irreversible pulpitis was characterized by spontaneous or lingering pain with deep caries and possible periodontal ligament changes. Pulpal necrosis was confirmed by absence of response to sensibility tests along with clinical and radiographic signs of periapical or furcal pathology. Given the limited reliability of sensibility tests in primary teeth, greater diagnostic emphasis was placed on clinical and radiographic findings. For teeth clinically diagnosed with irreversible pulpitis or pulpal necrosis and requiring pulp therapy, the diagnosis was confirmed during access cavity preparation. Presence of vital pulpal bleeding confirmed irreversible pulpitis, whereas absence of bleeding confirmed pulpal necrosis. This confirmatory procedure was performed only for teeth undergoing endodontic treatment and was not applicable to healthy or reversible pulpitis groups.

Pulpal oxygen saturation measurement

**Figure 2:**
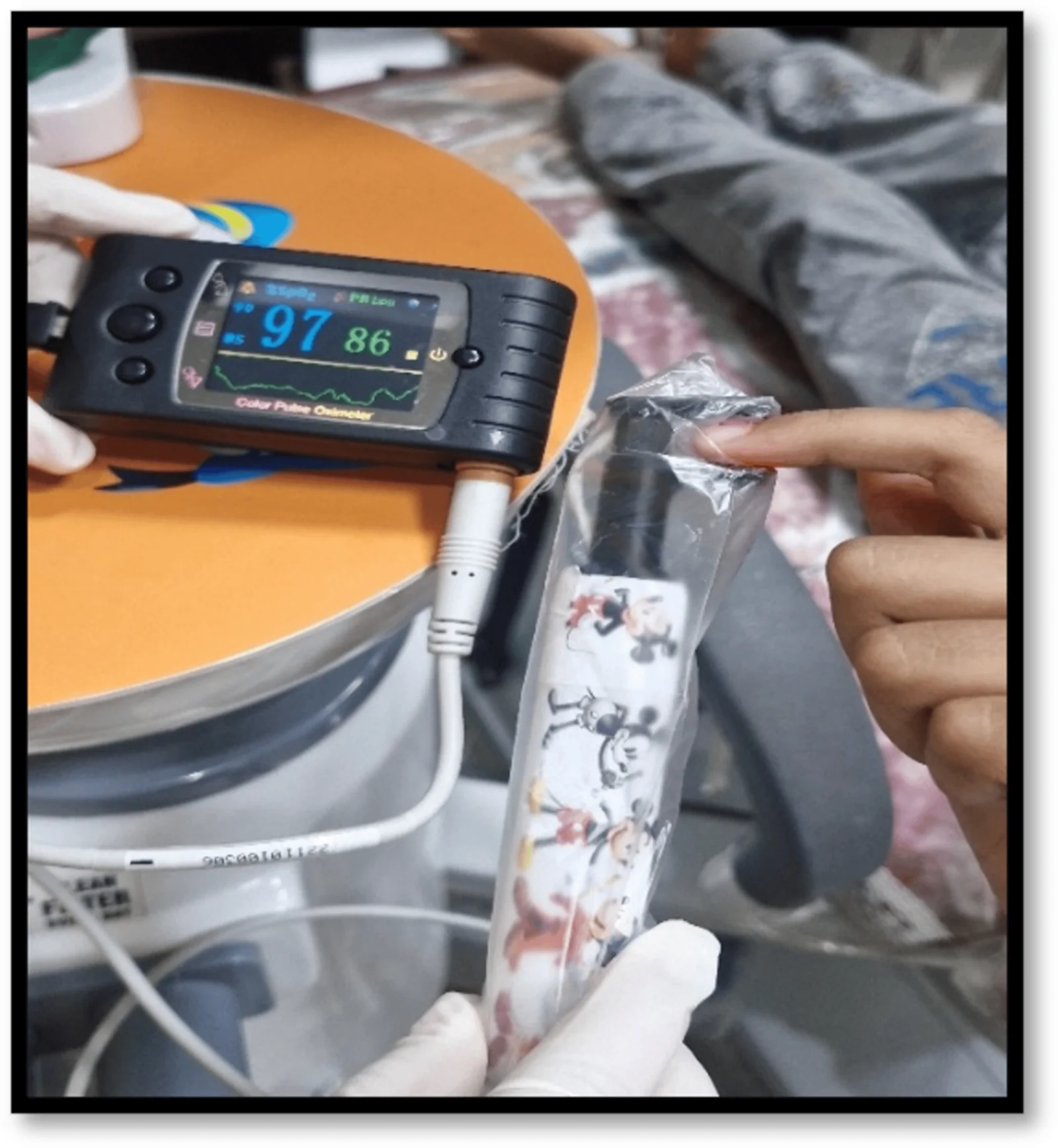
Measurement of finger oxygen saturation using pulse oximeter with customized dental probe holder

Device function was verified prior to each session by recording oxygen saturation from the participant's index finger, which also served as a systemic reference value (Figure 2).

**Figure 3:**
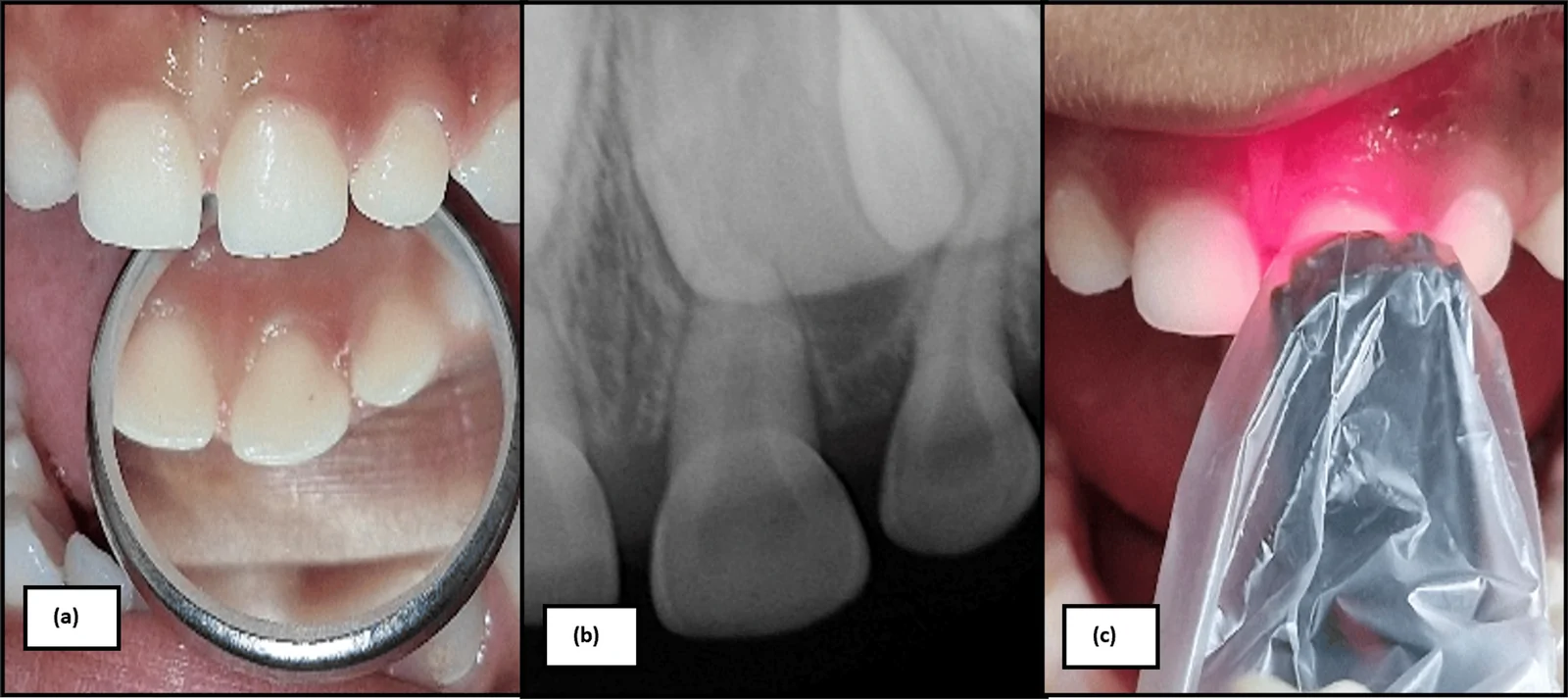
Selected tooth for the measurement of pulpal oxygen saturation (Group 1a Phase I) (a) Clinical photograph of the selected tooth in Group 1a Phase I; (b) Radiographic photograph of the selected tooth in Group 1a Phase I; (c) Measurement of pulp oxygen saturation of the selected tooth in Group 1a Phase I

**Figure 4:**
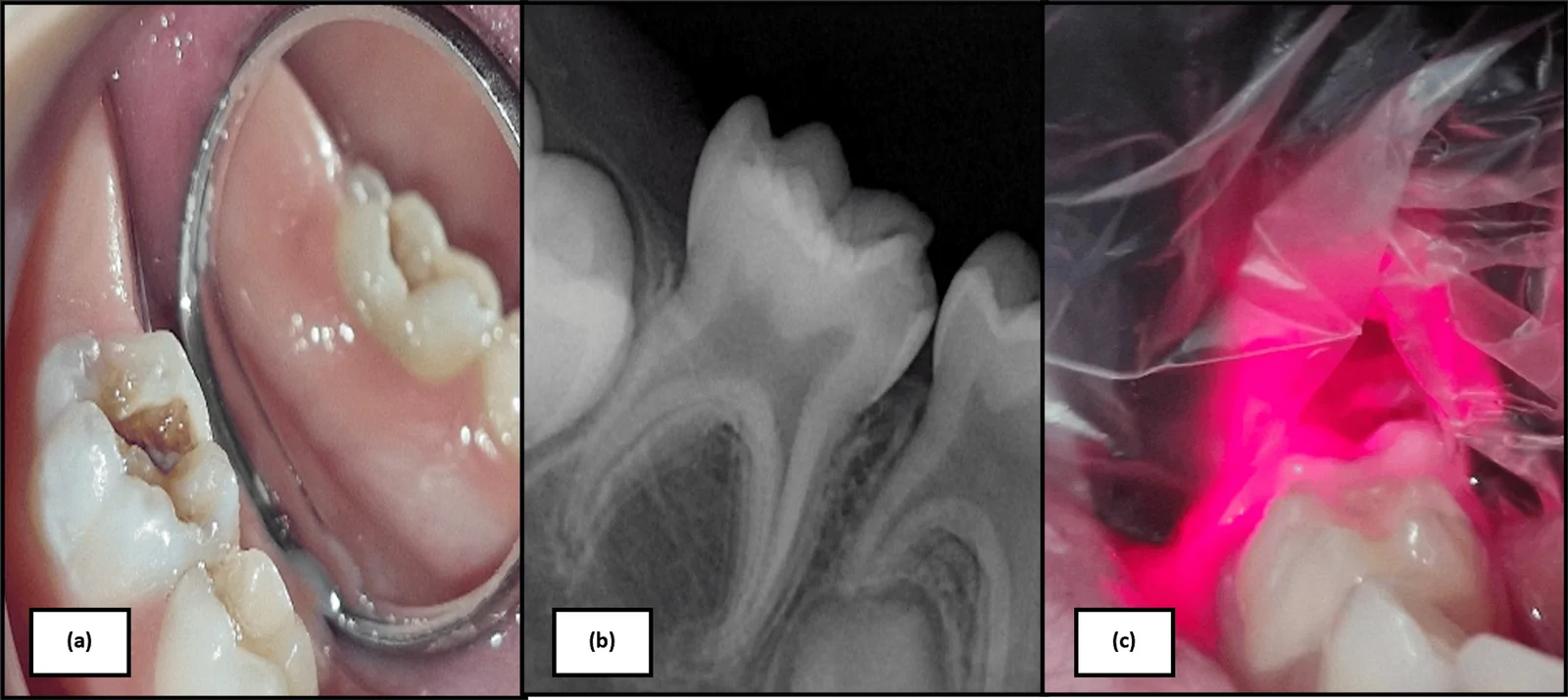
Selected tooth for the measurement of pulpal oxygen saturation (Group B Phase II) (a) Clinical photograph of the selected tooth in Group B Phase II; (b) Radiographic photograph of the selected tooth in Group B Phase II; (c) Measurement of pulp oxygen saturation of the selected tooth in Group B Phase II

The customized holder was seated on the selected tooth from phase I (Figure 3) and Phase II (Figure 4).

To minimize measurement variability, the probe was repositioned whenever unstable or fluctuating readings were observed until a stable signal was obtained. Three consecutive stable oxygen saturation readings were then recorded for each tooth, and the mean value was used for statistical analysis.

Statistical analysis

Data were analyzed using IBM SPSS Statistics software, version 26 (IBM Corp., Armonk, NY, USA). Normality was assessed using the Shapiro-Wilk test. Descriptive statistics were expressed as mean ± standard deviation. Intergroup comparisons among five tooth types were performed using one-way ANOVA or the Kruskal-Wallis test. Maxillary and mandibular comparisons were made using an independent t-test or Mann-Whitney test, as appropriate. Statistical significance was set at p < 0.05.

## Results

The study included children aged four to nine years evaluated across two phases. Gender distribution was largely balanced across all tooth groups, with only minor variations; a slight male predominance was observed in maxillary central and lateral incisors and near-equal distribution in mandibular tooth categories. Age-wise distribution corresponded with the eruption chronology of primary teeth, with mean ages ranging from 4.71 ± 0.68 years in maxillary central incisors to 7.29 ± 1.30 years in maxillary first molars, and from 4.75 ± 0.72 years to 6.59 ± 1.63 years in the mandibular arch. In Phase II, mean age ranged from 6.38 ± 1.59 years in the necrotic pulp group to 7.13 ± 1.31 years in endodontically treated molars, with an almost equal gender distribution across all pulpal condition groups.

Assessment of data distribution using the Shapiro-Wilk test revealed non-normal distribution of pulpal oxygen saturation values across all tooth types in both arches in Phase I, necessitating the use of non-parametric tests for inferential analysis. In Phase II, healthy pulp, reversible pulpitis, and irreversible pulpitis groups followed normal distribution, whereas the necrotic pulp group demonstrated significant deviation from normality. Finger oxygen saturation values remained consistently high and stable across all groups in both phases, ranging from 97.3% to 97.94%, with minimal variability and no statistically significant intergroup differences, confirming that systemic oxygen saturation did not confound the pulpal oxygen saturation measurements.

In Phase I, pulpal oxygen saturation values demonstrated a statistically significant difference across all tooth types in both arches (maxilla: χ² = 88.335, p < 0.001; mandible: χ² = 69.307, p < 0.001). In the maxillary arch, mean POS was highest in central incisors (89.29 ± 2.20%), followed by lateral incisors (88.46 ± 1.74%), canines (87.54 ± 1.89%), first molars (86.96 ± 2.16%), and second molars (85.09 ± 2.16%). A similar descending trend was observed in the mandibular arch, with central incisors recording the highest mean pulpal oxygen saturation (88.25 ± 1.83%), followed by lateral incisors (87.43 ± 1.92%), canines (86.54 ± 1.84%), first molars (85.82 ± 2.67%), and second molars (84.18 ± 2.81%). Across both arches, pulpal oxygen saturation values were consistently higher in anterior teeth compared to posterior teeth (Table 3).

**Table 3:** Comparison of pulpal oxygen saturation (%) among subjects in Phase I of the study Kruskal-Wallis test; *indicates a significant difference at p≤0.05.

Group	Subgroup	Mean	SD	Minimum	Maximum	χ^2^ value	p-value
Maxilla	Group 1a (Central incisor)	89.29	2.20	82	93	88.335	<0.001*
	Group 1b (Lateral incisor)	88.46	1.74	86	91		
	Group 1c (Canine)	87.54	1.89	85	90		
	Group 1d (First molar)	86.96	2.16	84	91		
	Group 1e (Second molar)	85.09	2.16	80	89		
Mandible	Group 2a (Central incisor)	88.25	1.83	85	92	69.307	<0.001*
	Group 2b (Lateral incisor)	87.43	1.92	84	91		
	Group 2c (Canine)	86.54	1.84	84	90		
	Group 2d (First molar)	85.82	2.67	80	90		
	Group 2e (Second molar)	84.18	2.81	78	89		

Comparison of pulpal oxygen saturation between anterior and posterior regions revealed a statistically significant difference in both the maxillary and mandibular arches. In the maxillary arch, anterior teeth recorded a significantly higher mean pulpal oxygen saturation (88.43 ± 2.07%) compared to posterior teeth (86.03 ± 2.35%) (z = −7.810, p < 0.001). Similarly, in the mandibular arch, anterior teeth demonstrated a higher mean pulpal oxygen saturation (87.40 ± 1.98%) compared to posterior teeth (85.00 ± 2.85%) (z = −6.841, p < 0.001). These findings consistently indicate that anterior primary teeth exhibit higher pulpal oxygen saturation than their posterior counterparts in both arches, reinforcing the anterior-to-posterior gradient in pulpal perfusion observed across the primary dentition (Table 4). 

**Table 4:** Comparison of pulpal oxygen saturation (%) in anterior and posterior teeth of maxillary and mandibular arches. Mann-Whitney test; *indicates a significant difference at p≤0.05.

Arch	Region	Mean	SD	Minimum	Maximum	z-value	p-value
Maxilla	Anterior	88.43	2.07	82	93	-7.810	<0.001*
	Posterior	86.03	2.35	80	91		
Mandible	Anterior	87.40	1.98	84	92	-6.841	<0.001*
	Posterior	85.00	2.85	78	90		

In Phase II, comparison of pulpal oxygen saturation across different pulpal conditions in primary molars revealed a statistically significant difference among all five groups (χ² = 72.226, p < 0.001). Healthy molars recorded the highest mean pulpal oxygen saturation (83.44 ± 2.56%), followed by reversible pulpitis (80.25 ± 2.70%), irreversible pulpitis (70.50 ± 3.98%), and necrotic pulp (11.13 ± 15.34%), while endodontically treated molars recorded a mean pulpal oxygen saturation of 0.00%, confirming complete absence of pulpal blood flow following treatment. A progressive and clinically meaningful decline in pulpal oxygen saturation was observed with advancing pulpal pathology, reflecting the deteriorating vascular status of the pulp across disease stages (Table 5). 

**Table 5:** Comparison of pulpal oxygen saturation (%) among molars with different pulpal conditions in Phase II study Kruskal-Wallis test; *indicates a significant difference at p≤0.05.

Group	Mean	SD	Minimum	Maximum	χ^2^ value	p-value
Group A (Healthy pulp)	83.44	2.56	78	87	72.226	<0.001*
Group B (Reversible pulpitis)	80.25	2.70	75	84		
Group C (Irreversible pulpitis)	70.50	3.98	62	78		
Group D (Necrotic pulp)	11.13	15.34	0	60		
Group E (Endodontically treated molars)	0.00	0.00	0	0		

Pairwise comparisons using the post hoc Bonferroni test revealed that healthy molars differed significantly from irreversible pulpitis (p = 0.005), necrotic pulp (p < 0.001), and endodontically treated molars (p < 0.001), but not from reversible pulpitis (p = 1.000), suggesting overlapping oxygen saturation ranges between healthy pulp and early inflammatory states. Reversible pulpitis differed significantly from necrotic pulp (p < 0.001) and endodontically treated molars (p < 0.001), but not from irreversible pulpitis (p = 0.206). Irreversible pulpitis showed a significant difference from endodontically treated molars (p = 0.004) but not from necrotic pulp (p = 0.148), indicating convergence of oxygen saturation values in advanced pulpal disease. Necrotic pulp and endodontically treated molars showed no significant difference from each other (p = 1.000), both reflecting absent or near-absent pulpal perfusion (Table 6).

**Table 6:** Pairwise comparisons of pulpal oxygen saturation (%) among different pulpal conditions in mandibular primary molars. Dunn's post hoc pairwise comparison with Bonferroni adjustment; *indicates a significant difference at p≤0.05.

Tooth	Maxilla
Healthy molars vs Reversible pulpitis	1.000
Healthy molars vs Irreversible pulpitis	0.005*
Healthy molars vs Necrotic pulp	<0.001*
Healthy molars vs Endodontically treated molars	<0.001*
Reversible pulpitis vs Irreversible pulpitis	0.206
Reversible pulpitis vs Necrotic pulp	<0.001*
Reversible pulpitis vs Endodontically treated molars	<0.001*
Irreversible pulpitis vs Necrotic pulp	0.148
Irreversible pulpitis vs Endodontically treated molars	0.004*
Necrotic pulp vs Endodontically	1.000

## Discussion

Accurate assessment of pulp vitality is essential for diagnosis and treatment planning in pediatric dentistry. The American Academy of Pediatric Dentistry (AAPD) [9] states that thermal and electric pulp tests are unreliable in primary and immature permanent teeth because they assess neural response rather than pulpal vascularity. Pulse oximetry offers an objective, non-invasive assessment of pulpal blood oxygen saturation, making it a promising alternative in children [4]. However, evidence regarding its use in primary teeth remains limited, with anatomical differences between anterior and posterior teeth and probe adaptation challenges restricting the generalization and clinical application of earlier findings. In the present study, standardized sensor placement using a customized dental holder, averaging three readings, and recording systemic oxygen saturation improved measurement reliability, while bleeding on access cavity preparation served as the reference standard to validate pulpal vitality. The study further demonstrated that pulpal oxygen saturation varies according to tooth type, arch, and pulpal status, providing tooth-specific reference values for primary dentition.

The present study demonstrated a consistent anterior-to-posterior decline in pulpal oxygen saturation in both arches, with central incisors recording the highest values (maxilla: 89.29 ± 2.20%; mandible: 88.25 ± 1.83%) and second molars the lowest (maxilla: 85.09 ± 2.16%; mandible: 84.18 ± 2.81%). This pattern is consistent with Igna et al., who similarly reported higher pulpal oxygen saturation in anterior primary and permanent teeth compared with posterior teeth [10]. Pairwise comparisons further showed that while anterior teeth differed significantly from posterior teeth overall, adjacent tooth types such as lateral incisors and canines, or canines and first molars, did not differ significantly from one another, indicating that the decline in pulpal oxygen saturation progresses gradually across the arch rather than abruptly. This gradient is best explained anatomically: anterior teeth have thinner enamel-dentin thickness and a more direct optical pathway, allowing greater light transmission, whereas posterior teeth possess thicker hard tissue and more complex pulp chamber anatomy that increase light scattering and lower the measured pulpal oxygen saturation independent of true vascular status. These findings support the use of tooth-specific reference ranges rather than a single universal cut-off for pulse oximetry in primary teeth.

Maxillary primary teeth also demonstrated significantly higher pulpal oxygen saturation than their mandibular counterparts for central incisors, lateral incisors, canines, and first molars, although second molars showed no significant inter-arch difference. A systematic review similarly noted variation in oxygen saturation between maxillary and mandibular teeth attributable to differences in crown anatomy and optical path length, and Estrela et al. reported comparable inter-arch differences in maxillary and mandibular molars, reinforcing the influence of arch-related anatomical factors on pulse oximetry readings [11,12]. Beyond anatomy, this difference is also plausibly vascular in origin. Maxillary teeth receive blood supply through multiple branches of the superior alveolar arteries, forming a richer collateral network, whereas mandibular teeth are supplied primarily by a single source, the inferior alveolar artery. Using laser Doppler flowmetry, Guo et al. similarly demonstrated greater vascular responsiveness and pulpal blood flow in maxillary compared with mandibular teeth, supporting this vascular explanation for the higher maxillary pulpal oxygen saturation values observed in the present study [13]. The absence of a significant inter-arch difference in second molars may reflect comparable tissue thickness and structural complexity at this site in both arches, producing similar optical and circulatory conditions during measurement.

In Phase II, pulpal oxygen saturation declined progressively across the spectrum of pulpal disease, from healthy pulp (83.44 ± 2.56%) through reversible pulpitis (80.25 ± 2.70%) and irreversible pulpitis (70.50 ± 3.98%) to pulpal necrosis (11.13 ± 15.34%), with endodontically treated molars showing complete absence of saturation (0.00%). Pairwise comparison showed that healthy pulp did not differ significantly from reversible pulpitis, indicating that pulpal vascularity is largely preserved in early inflammation, whereas pulpal oxygen saturation declined significantly once inflammation progressed to irreversible pulpitis and beyond, consistent with rising intrapulpal pressure, vascular congestion, and eventual microcirculatory failure. Necrotic and endodontically treated teeth were statistically indistinguishable, both reflecting the complete absence of pulpal perfusion. The relatively wide range of pulpal oxygen saturation values observed in the necrotic pulp group, including an isolated higher reading, may be attributable to residual vascular tissue, signal contamination from surrounding periodontal tissues, measurement variability, or the inherent challenges associated with diagnosing pulpal necrosis in primary teeth. This progressive pattern parallels the findings of Setzer et al., who reported a similar stepwise decline in oxygen saturation with advancing pulpal pathology in permanent teeth [14], and of Anusha et al., who found significantly higher saturation in healthy teeth with a gradual reduction across increasing pulpal pathology and complete absence in endodontically treated teeth [15]. Together, these findings support the ability of pulse oximetry to objectively differentiate stages of pulpal disease, offering a vascularly grounded alternative to the subjective sensory responses on which conventional sensibility tests depend.

Collectively, the present findings demonstrate that pulpal oxygen saturation in primary teeth is tooth-specific and varies according to tooth type, arch, region, and pulpal status, with a progressive decline corresponding to advancing pulpal pathology. The study establishes clinically relevant baseline pulpal oxygen saturation values for different primary tooth types and supports pulse oximetry, when used with a customized probe holder and standardized measurement protocol, as an objective, noninvasive, and reliable adjunct to conventional pulp sensibility tests for assessing pulp vitality in children. The use of bleeding on access cavity preparation as the reference standard further strengthens the validity of the findings. However, the study was limited by its cross-sectional design, inclusion of children aged four to nine years, and assessment of diseased pulps only in mandibular molars. 

The present study has certain limitations. The use of a convenience sampling method and a single-center design may limit the generalizability of the findings. Although all measurements were performed using a standardized protocol, formal examiner calibration, assessment of intra-examiner reproducibility, and statistical evaluation of the reproducibility of the customized pulse oximeter holder (e.g., intraclass correlation coefficient) were not performed. In addition, multiple teeth were included from some participants, and the analyses were conducted at the tooth level. Furthermore, Phase II was limited to mandibular primary molars. Future multicenter longitudinal studies incorporating validated measurement protocols and appropriate statistical methods are warranted to strengthen the evidence and further establish the clinical utility of pulse oximetry in primary dentition.

## Conclusions

Pulse oximetry demonstrated promising potential as an objective and non-invasive adjunctive method for assessing pulp vitality in primary teeth. The present study showed that pulpal oxygen saturation varied significantly according to tooth type, dental arch, anatomical region, and pulpal status. Healthy primary teeth exhibited tooth-specific oxygen saturation values, with higher readings in anterior than posterior teeth and in the maxillary arch compared with the mandibular arch. Furthermore, pulpal oxygen saturation generally declined with increasing severity of pulpal disease, although some overlap was observed between certain pulpal conditions. The customized pulse oximeter holder facilitated standardized probe positioning across different primary tooth morphologies, supporting consistent measurement acquisition. These findings suggest that pulse oximetry may complement conventional pulp sensibility tests by providing an objective assessment of pulpal vascular status. Further multicenter longitudinal studies involving larger and more diverse populations are warranted to validate tooth-specific reference values and further evaluate the clinical utility of pulse oximetry in pediatric endodontic practice.
